# Reactance to Social Authority in a Sugar Reduction Informational Video: Web-Based Randomized Controlled Trial of 4013 Participants

**DOI:** 10.2196/29664

**Published:** 2021-11-22

**Authors:** Violetta Hachaturyan, Maya Adam, Caterina Favaretti, Merlin Greuel, Jennifer Gates, Till Bärnighausen, Alain Vandormael

**Affiliations:** 1 Heidelberg Institute of Global Health University of Heidelberg Heidelberg Germany; 2 Department of Pediatrics Stanford University School of Medicine Stanford, CA United States; 3 Icahn School of Medicine at Mount Sinai New York, NY United States; 4 Africa Health Research Institute Durban South Africa; 5 Harvard Center for Population and Development Studies Cambridge, MA United States

**Keywords:** sugar reduction, reactance, animated video, digital intervention, health communication

## Abstract

**Background:**

Short and animated story-based (SAS) videos can be an effective strategy for promoting health messages. However, health promotion strategies often motivate the rejection of health messages, a phenomenon known as reactance. In this study, we examine whether the child narrator of a SAS video (perceived as nonthreatening, with low social authority) minimizes reactance to a health message about the consumption of added sugars.

**Objective:**

This study aims to determine whether our SAS intervention video attenuates reactance to the sugar message when compared with a content placebo video (a health message about sunscreen) and a placebo video (a nonhealth message about earthquakes) and determine if the child narrator is more effective at reducing reactance to the sugar message when compared with the mother narrator (equivalent social authority to target audience) or family physician narrator (high social authority) of the same SAS video.

**Methods:**

This is a web-based randomized controlled trial comparing an intervention video about sugar reduction narrated by a child, the child’s mother, or the family physician with a content placebo video about sunscreen use and a placebo video about earthquakes. The primary end points are differences in the antecedents to reactance (proneness to reactance, threat level of the message), its components (anger and negative cognition), and outcomes (source appraisal and attitude). We performed analysis of variance on data collected (N=4013) from participants aged 18 to 59 years who speak English and reside in the United Kingdom.

**Results:**

Between December 9 and December 11, 2020, we recruited 38.62% (1550/4013) men, 60.85% (2442/4013) women, and 0.52% (21/4013) others for our study. We found a strong causal relationship between the persuasiveness of the content promoted by the videos and the components of reactance. Compared with the placebo (mean 1.56, SD 0.63) and content placebo (mean 1.76, SD 0.69) videos, the intervention videos (mean 1.99, SD 0.83) aroused higher levels of reactance to the message content (*P*<.001). We found no evidence that the child narrator (mean 1.99, SD 0.87) attenuated reactance to the sugar reduction message when compared with the physician (mean 1.95, SD 0.79; *P*=.77) and mother (mean 2.03, SD 0.83; *P*=.93). In addition, the physician was perceived as more qualified, reliable, and having more expertise than the child (*P<*.001) and mother (*P<*.001) narrators.

**Conclusions:**

Although children may be perceived as nonthreatening messengers, we found no evidence that a child narrator attenuated reactance to a SAS video about sugar consumption when compared with a physician. Furthermore, our intervention videos, with well-intended goals toward audience health awareness, aroused higher levels of reactance when compared with the placebo videos. Our results highlight the challenges in developing effective interventions to promote persuasive health messages.

**Trial Registration:**

German Clinical Trials Registry DRKS00022340; https://tinyurl.com/mr8dfena

**International Registered Report Identifier (IRRID):**

RR2-10.2196/25343

## Introduction

### Background

Digital health interventions that promote educational messages to improve knowledge and change behaviors are commonly used as effective health promotion strategies. Comparisons of digital behavior change interventions with traditional face-to-face interventions indicate that web-based health promotion is generally at least as effective as conventional approaches and has several advantages, such as low cost, feasibility, and scalability [1,2]. Existing evidence suggests that the use of pictures [3], entertainment education [4], digital storytelling, and narrative structured messages [4] are some of the successful approaches for creating compelling, evidence-based health messages. As narrative messages do not include a direct, controlling language and words, such as *should*, *must*, and *required* [5,6], and conceal the persuasive intent, they can be more effective when compared with traditional health communication strategies [7]. To further explore the effectiveness of these innovative strategies in health communication, we created a short and animated story-based (SAS) video that draws from entertainment-education media, communication theory, and the animated entertainment industry to promote healthy behaviors over social media channels [8]. However, SAS videos may face the same challenges faced by other traditional methods of health persuasion that often arouse a motivation to reject the health message, a phenomenon known as reactance [9].

The theory of reactance consists of 4 elements: (1) perceived freedom, which individuals possess insofar as they are aware of it and can enact it; (2) threat to freedom, when pressure is exerted that makes it difficult to enact that freedom; (3) reactance, which refers to the motivation to reestablish the threatened freedom; and (4) direct restoration, which involves the freedom of the individual to perform the forbidden behavior [5]. Reactance plays a critical role in determining the effectiveness and acceptance of health promotion interventions. This has led to an active research agenda in developing strategies to reduce reactance in several areas, such as e-cigarette use [10], littering [11], alcohol [12], and eating behaviors [13], among others [14-16].

### Objectives

In this study, we produced a SAS video with a message about reducing the consumption of added sugars. Designed for a diverse and global audience, the animated video uses a narrative based structure to minimize reactance to the sugar message. For our first hypothesis, we assess if our narrative-based, animated video is effective at attenuating reactance to a persuasive health message.

We hypothesize that there is a causal pathway between exposure to a SAS video about sugar intake reduction and reactance, its antecedents, and outcomes.

Hypothesis 1

While designing the video, we considered the degree of social authority that should be assigned to the narrator. First, we selected the traditional role of a physician who has high social authority. Although health experts and physicians are often used to promote health messages [17-19], previous studies have shown that individuals may perceive these messengers as coercive, threatening, or having a hidden persuasive intent [20], which could sustain or heighten reactance [9,21]. Therefore, we considered a child narrator as a potentially powerful and effective narrator, as a child may be perceived as nonthreatening, neutral, and without having an ulterior motive. To date, we were unable to find prior research on the effectiveness of a child narrator to attenuate reactance using a narrative-based, animated video format. The second hypothesis is as follows:

We hypothesize that a SAS video about sugar consumption narrated by a child (low social authority) will arouse less reactance when compared with a video narrated by the child’s mother (equivalent social authority to the target audience) or the family doctor (high social authority).

Hypothesis 2

We used a randomized controlled trial (RCT) to measure the causal effect of social authority on reactance to a short, animated video about sugar intake reduction. The randomization ensures that there are no systematic differences introduced at the enrollment stage, which may lead to potential bias. In addition, an innovative feature of our study is the use of 2 placebo groups, which enabled us to isolate the health awareness and content effects of the intervention video.

## Methods

### Trial Design

This study is a web-based RCT with 3 intervention arms (*arms 1-3*), a content placebo arm (*arm 4*), and a placebo arm (*arm 5*). The participants in each intervention arm watched the same sugar video narrated by a child (*arm 1:* low social authority), the child’s mother (*arm 2:* equivalent social authority), or the physician (*arm 3:* high social authority). *Arm 4* watched a content placebo video with a health message about tanning and sunscreen (no sugar message), and *arm 5* watched a placebo video about earthquakes (no sugar or health message).

### Participants

We used the Prolific platform (Prolific Academic Ltd) [22] to recruit the study participants. Prolific is a web-based platform designed to connect researchers and individuals from different countries interested in participating in web-based academic research studies in exchange for payment. The main advantages of the platform are access to a diverse pool of web-based participants, affordability, and speed of recruitment [23]. Prolific implements several tools to reduce selection biases and allows researchers to specify various recruitment criteria, such as first language, age, sex, country of residence, and ethnicity, among others. Currently, the platform’s participant pool consists of 150,000 individuals from 34 countries. Inclusion criteria in our study included being between the ages of 18 and 59 years (male, female, or other), being able to speak English, and having a residence in the United Kingdom. Exclusion criteria were not any of the inclusion criteria. The participants were not excluded based on an existing health condition (eg, diabetes) because Prolific does not collect health information from its users. Participants were provided with an informed consent form on the Prolific platform, which explained the purpose of the study, the risks and benefits of the research, and the means by which a participant could contact the researcher (and the human subjects review board at the Heidelberg University). After consenting, the Prolific platform redirected participants to the Gorilla platform (Cauldron Science Limited) [24], where the study was hosted. Gorilla is a cloud platform that provides versatile tools for web-based, experimental, and behavioral research [25]. The participants were also informed that they would be paid £1 (US $1.37) for the 10-minute completion time. We recruited participants until the target sample size was reached.

### Procedures

Participants were asked basic demographic questions about their age, sex, and highest education level. The Gorilla algorithm then randomly assigned participants at a 1:1:1:1:1 ratio to the trial arms. The participants watched 1 video from start to finish.

The intervention video (*arms 1-3*) is a SAS video about sugar intake reduction developed by our coauthor (MA) at the Stanford School of Medicine [26-28]. It is animated, completely in English, and 3.42 minutes long. The video includes 2 main characters: a mother and her preadolescent daughter who are engaging in food-related activities, such as grocery shopping and cooking dinner. The video presents educational content on sugar-related health problems and includes a review of the World Health Organization recommendations for the daily consumption of added sugars. The narrative also mentions the girl’s father who dies from diabetes complications because of frequent consumption of soda drinks.

The content placebo video is similar in style to the sugar intervention video—it is animated, has a length of 3.42 minutes, and a health message about the use of sunscreen and tanning [29]. We used the content placebo video to isolate the *content effect* of the sugar intervention video. As both the intervention and content placebo videos have a health message, we expect that any significant difference in reactance should be due to the sugar reduction content of the intervention video.

The placebo video [30] is also animated and has the same length as the intervention and content placebo videos. It describes the causes and characteristics of earthquakes, and contains no health-related or sugar consumption content. As the content placebo video promotes a health message and the placebo video does not, we expected the placebo video to arouse a very small (or even null) level of reactance. Thus, any significant difference in reactance levels between the content placebo and the placebo videos after randomization can be attributed to the content of the sunscreen message. We call this difference the *health awareness effect*. We describe the *total intervention effect* as the difference between the sugar intervention and the placebo videos, which is the sum of the content and health awareness effects.

The full explanation for the choice of comparators has been described in the study protocol [31].

### Outcome Measures

**Figure 1:**
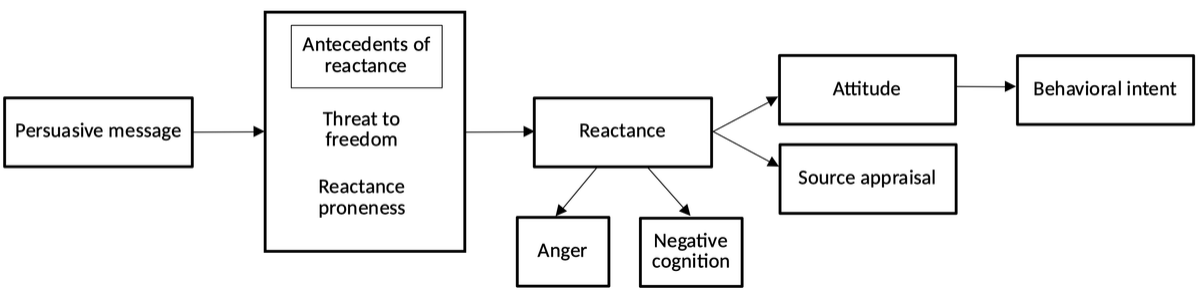
The Intertwined Process Cognitive-Affective Model, adapted from Dillard and Shen [5] and Zhang [32].

The primary outcomes in this study were based on the Intertwined Process Cognitive-Affective Model from Dillard and Shen [5] and Zhang [32] (Figure 1). In this model, there are 2 antecedents to reactance (threat to freedom and trait proneness to reactance), reactance itself (consisting of anger and negative cognition), and its consequences (source appraisal, attitude, and behavioral intent). Reactance serves as a mediator between the antecedents of reactance and the behavioral intent to undertake the promoted health activity. In this paper, we focus on the antecedents of reactance (trait reactance proneness and threat to freedom), psychological reactance (consisting of anger and negative cognition), source appraisal, and attitude. All items were measured on a 5-point Likert scale (unless stated otherwise) with the following points: (1) strongly disagree, (2) disagree, (3) neither agree nor disagree, (4) agree, and (5) strongly agree.

### Trait Reactance Proneness

Trait reactance proneness refers to reactance being a personality attribute that causes the levels of experienced reactance to vary from individual to individual [33]. High-trait reactant individuals tend to experience reactance in certain situations and are more resistant to persuasion due to their strong need for independence and autonomy and a tendency to oppose authority [5,34].

Trait reactance proneness in this study was measured using the Hong Psychological Reactance Scale developed by Hong et al [33]. The scale consists of 11 items that comprise 4 major factors: emotional response to restricted choice, reactance to compliance, resisting influence from others, and reactance to advice and recommendations (Textbox 1).

**Trait reactance items based on the Hong Psychological Reactance Scale [33].**

**Emotional response to restricted choice**

- 6. I become frustrated when I am unable to make free and independent decisions.
- 7. It irritates me when someone points out things which are obvious to me.
- 8. I become angry when my freedom of choice is restricted.

**Reactance to compliance**

- 1. Regulations trigger a sense of resistance in me.
- 2. I find contradicting others stimulating.
- 3. When something is prohibited, I usually think, “That’s exactly what I am going to do.”

**Resisting influence from others**

- 11. I resist the attempts of others to influence me.
- 12. It makes me angry when another person is held up as a role model for me to follow.
- 13. When someone forces me to do something, I feel like doing the opposite.

**Reactance to advice and recommendations**

- 5. I consider advice from others to be an intrusion.
- 9. Advice and recommendations usually induce me to do just the opposite.

### Threat to Freedom

To measure the threat level of the message, we used the following 4 items from Dillard and Shen [5]:

1. The message threatened my freedom to choose.
2. The message tried to make a decision for me.
3. The message tried to manipulate me.
4. The message tried to pressure me.

### Psychological Reactance

Following Dillard and Shen’s model, psychological reactance consists of 2 major components: (1) affective (anger) and (2) cognitive (negative cognition) [5]. Therefore, reactance was assessed by measuring the average of all items on the anger and negative cognition scales. To measure anger, the following 4 items were used:

1. This message makes me feel irritated.
2. This message makes me feel annoyed.
3. This message makes me feel aggravated.
4. This message makes me feel angry.

Negative cognition was measured using the scale from Quick et al [35] with the following 3 items:

1. The thoughts I had while watching this video were mostly unfavorable.
2. The thoughts I had while watching this video were mostly negative.
3. The thoughts I had while watching this video were mostly bad.

### Source Appraisal

Source appraisal, also called source derogation [6], refers to the audience’s evaluation of the source of the message. Source appraisal was examined using the question “The narrator of this video was...” and 7 semantic differential items anchored on either end with opposing adjectives: stupid or smart, unknowledgeable or knowledgeable, uninformed or informed, unintelligent or intelligent, unqualified or qualified, unreliable or reliable, and inexpert or expert [36]. The category ratings were scored from 1 to 5, with higher scores indicating more favorable evaluations of the message source (reverse-coded).

### Attitude

Attitude toward message advocacy was measured using the following 4 items from Shen [37]:

1. I agree with what the message recommends.
2. I support what the message advocates.
3. I am in favor of the position in the message.
4. I endorse the claims made in the message.

### Sample Size

We calculated the sample size needed for pairwise comparisons among the 5 groups using the analysis of variance. Our calculations resulted in a sample size of n=769 per group [31]. For a 5-way comparison, the sample size is N=3845. We selected a sample size of N=4000 to ensure we have sufficient power and account for attrition.

### Statistical Methods

Descriptive statistics were used to obtain means and SDs of the demographic data of the sample, which included age, sex, and education status. We used analysis of variance to estimate the difference in the means of the outcome measures between the sugar intervention videos, the content placebo video, and the placebo video. The significance level α was set at .05. Post hoc tests with Tukey range method were used to create CIs for all pairwise differences between the means while controlling for family error rate. The placebo arm was chosen as the reference group, as the placebo video did not include any content related to sugar or health and, therefore, did not have any persuasive intent. All statistical analyses were performed using the statistical software R (R Foundation for Statistical Computing).

### Availability of Data and Materials

The data were collected and stored on the Gorilla platform. The study investigators own and have complete control of the research data, which can be accessed at any time. For statistical analysis, the data were downloaded and stored safely in a computing system maintained by the Heidelberg University.

### Ethical Approval

Ethical approval was obtained from the Heidelberg University’s ethics committee (Universität Heidelberg Ethikkommission der Medizinische Fakultät) on March 18, 2020, protocol: S-088/2020.

## Results

### Sample Characteristics

**Figure 2:**
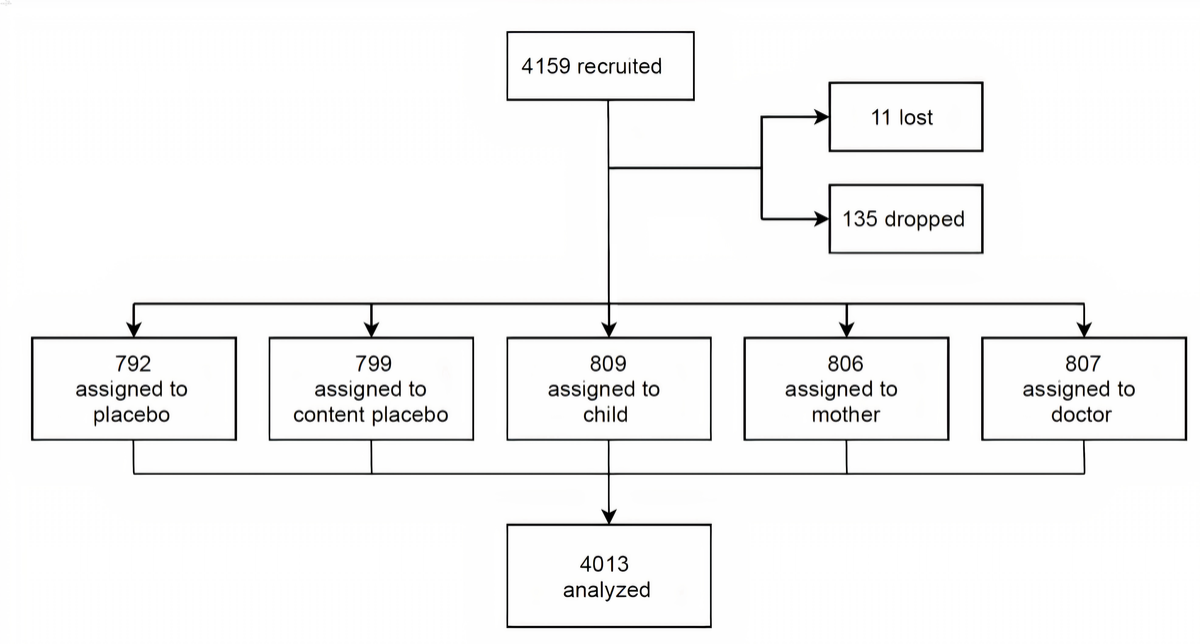
Trial design.

Between December 9, 2020, and December 11, 2020, a total of 4159 participants from the United Kingdom were recruited for the trial. After recruitment, 0.26% (11/4159) participants were lost and another 3.24% (135/4159) participants were dropped, as they did not complete the study for either technical reasons (poor internet connection, video loading issues, system crash, and so on) or other unknown reasons. Of the recruited sample, 96.48% (4013/4159) completed the trial and were included in the final analysis (Figure 2). Table 1 provides the demographic characteristics of the participants by group, including gender, age, and education level. Of the sample, 60.90% (2444/4013) were female, 32.27% (1295/4013) were aged between 25 and 34 years, and 64.09% (2572/4013) had some college education or a bachelor’s degree. There were no significant differences in baseline characteristics between the 5 arms, suggesting that the randomization was efficient.

**Table 1:** Summary of demographic characteristics by group (N=4013).

Characteristics			Placebo (n=792), n (%)		Content placebo (n=799), n (%)		Child voice (n=809), n (%)		Mother voice (n=806), n (%)	Physician voice (n=807), n (%)		*P* value	
**Gender**													.83
	Female	485 (61.24)		481 (60.20)		494 (61.06)		485 (60.17)		497 (61.59)			
	Male	300 (37.88)		313 (39.17)		313 (38.69)		317 (39.33)		307 (38.04)			
	Other	7 (0.88)		5 (0.63)		2 (0.25)		4 (0.50)		3 (0.37)			
**Age (years)**													.96
	18-24	208 (26.26)		184 (23.03)		214 (26.45)		200 (24.81)		195 (24.16)			
	25-34	250 (31.57)		259 (32.42)		266 (32.88)		267 (33.13)		254 (31.47)			
	35-44	167 (21.09)		175 (21.90)		175 (21.63)		167 (20.72)		190 (23.54)			
	45-54	120 (15.15)		130 (16.27)		109 (13.47)		121 (15.01)		120 (14.87)			
	55-59	47 (5.93)		51 (6.38)		45 (5.56)		51 (6.33)		48 (5.95)			
**Education**													.97
	Primary School or less	11 (1.39)		13 (1.63)		8 (0.99)		9 (1.12)		10 (1.24)			
	Completed High School	126 (15.91)		123 (15.39)		117 (14.46)		131 (16.25)		126 (15.61)			
	Some College, Bachelor’s Degree	500 (63.13)		501 (62.70)		530 (65.51)		518 (64.27)		525 (65.06)			
	Master’s Degree, Doctorate	155 (19.57)		162 (20.27)		154 (19.04)		148 (18.36)		146 (18.09)			

### Outcome Measures

Table 2 presents the descriptive statistics, including the mean and SDs of all the key variables measured in this study.

**Table 2:** Mean and SD of outcome variables in study arms.

Characteristics		Placebo (n=792), mean (SD)	Content placebo (n=799), mean (SD)	Child voice (n=809), mean (SD)	Mother voice (n=806), mean (SD)	Physician voice (n=807), mean (SD)	*P* value
Trait reactance proneness		2.98 (0.48)	2.97 (0.51)	2.99 (0.52)	2.97 (0.54)	2.97 (0.52)	.92
Threat to freedom		1.46 (0.55)	1.83 (0.68)	2.28 (0.86)	2.34 (0.82)	2.20 (0.80)	<.001
**Psychological reactance**		1.56 (0.63)	1.76 (0.69)	1.99 (0.87)	2.03 (0.83)	1.95 (0.79)	<.001
	Anger	1.51 (0.63)	1.70 (0.72)	1.95 (0.90)	1.98 (0.87)	1.90 (0.83)	<.001
	Negative cognition	1.64 (0.77)	1.84 (0.79)	2.05 (0.95)	2.09 (0.91)	2.02 (0.86)	<.001
Attitude		3.79 (0.60)	4.28 (0.60)	4.22 (0.64)	4.14 (0.64)	4.18 (0.65)	<.001
**Source appraisal**		3.91 (0.52)	3.67 (0.52)	3.57 (0.58)	3.63 (0.56)	3.72 (0.54)	<.001
	Stupid or smart	3.77 (0.78)	3.50 (0.79)	3.74 (0.81)	3.59 (0.80)	3.62 (0.75)	<.001
	Unknowledgeable or knowledgeable	4.06 (0.71)	3.93 (0.75)	3.82 (0.81)	3.86 (0.74)	3.88 (0.67)	<.001
	Uninformed or informed	4.17 (0.59)	4.08 (0.66)	4.03 (0.69)	4.02 (0.68)	4.03 (0.62)	<.001
	Unintelligent or intelligent	3.96 (0.63)	3.69 (0.67)	3.80 (0.67)	3.72 (0.66)	3.72 (0.65)	<.001
	Unqualified or qualified	3.75 (0.71)	3.44 (0.68)	3.06 (0.94)	3.34 (0.74)	3.56 (0.72)	<.001
	Unreliable or reliable	3.91 (0.67)	3.69 (0.62)	3.57 (0.75)	3.64 (0.72)	3.75 (0.66)	<.001
	Inexpert or expert	3.71 (0.71)	3.40 (0.65)	2.99 (0.86)	3.23 (0.73)	3.45 (0.69)	<.001

### Antecedents of Reactance

**Figure 3:**
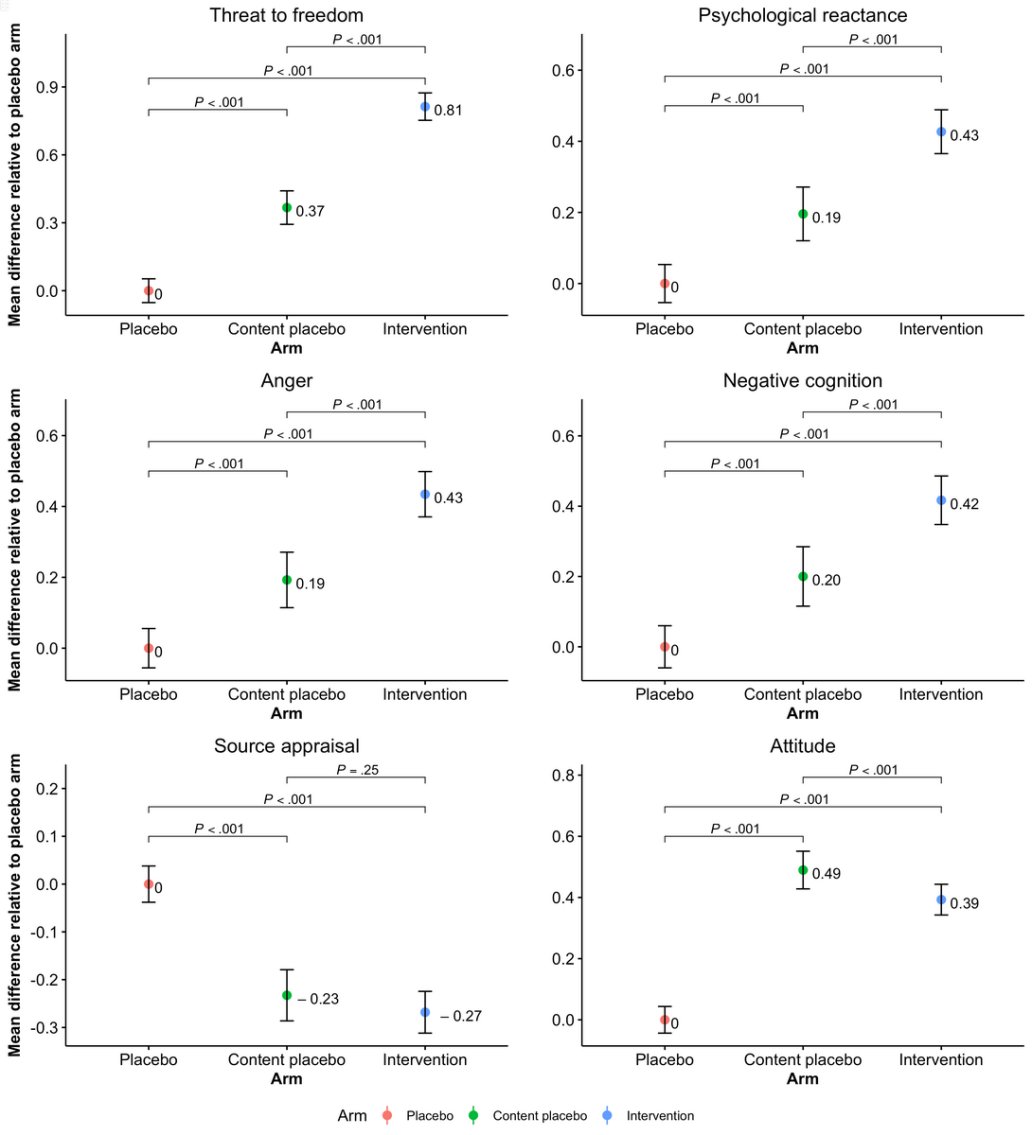
Mean differences in outcome measures among the placebo arm (reference arm), content placebo, and intervention arms. *P* values represent the significance of the observed difference in means among the study arms.

Trait reactance proneness and threat to freedom are antecedents to reactance. Higher scores on trait proneness and threat to freedom scales indicate higher proneness to reactance and greater perceived threat, respectively. As shown in Table 2, the mean scores for trait reactance proneness in the 5 arms were in the mean range 2.97-2.99 (SD 0.48-0.52) with *P*=.92, which did not vary significantly between the 5 arms. When comparing the means scores for threat to freedom, the analysis revealed a significant difference between the 5 groups (*P*<.001). Furthermore, the pairwise comparisons for the threat level, using a Bonferroni correction, indicated that participants in the content placebo and intervention arms reported higher threat to freedom when compared with the placebo arm (*P*<.001; Figure 3). When comparing intervention arms between each other, participants in the mother arm indicated a higher threat level than those in the doctor arm (*P*=.002) but not in the child arm (*P*=.52). Although the threat level in the child arm (mean 2.28, SD 0.86) was slightly higher than in the doctor arm (mean 2.20, SD 0.80), the difference was not found to be significant (*P*=.21).

### Psychological Reactance

**Figure 4:**
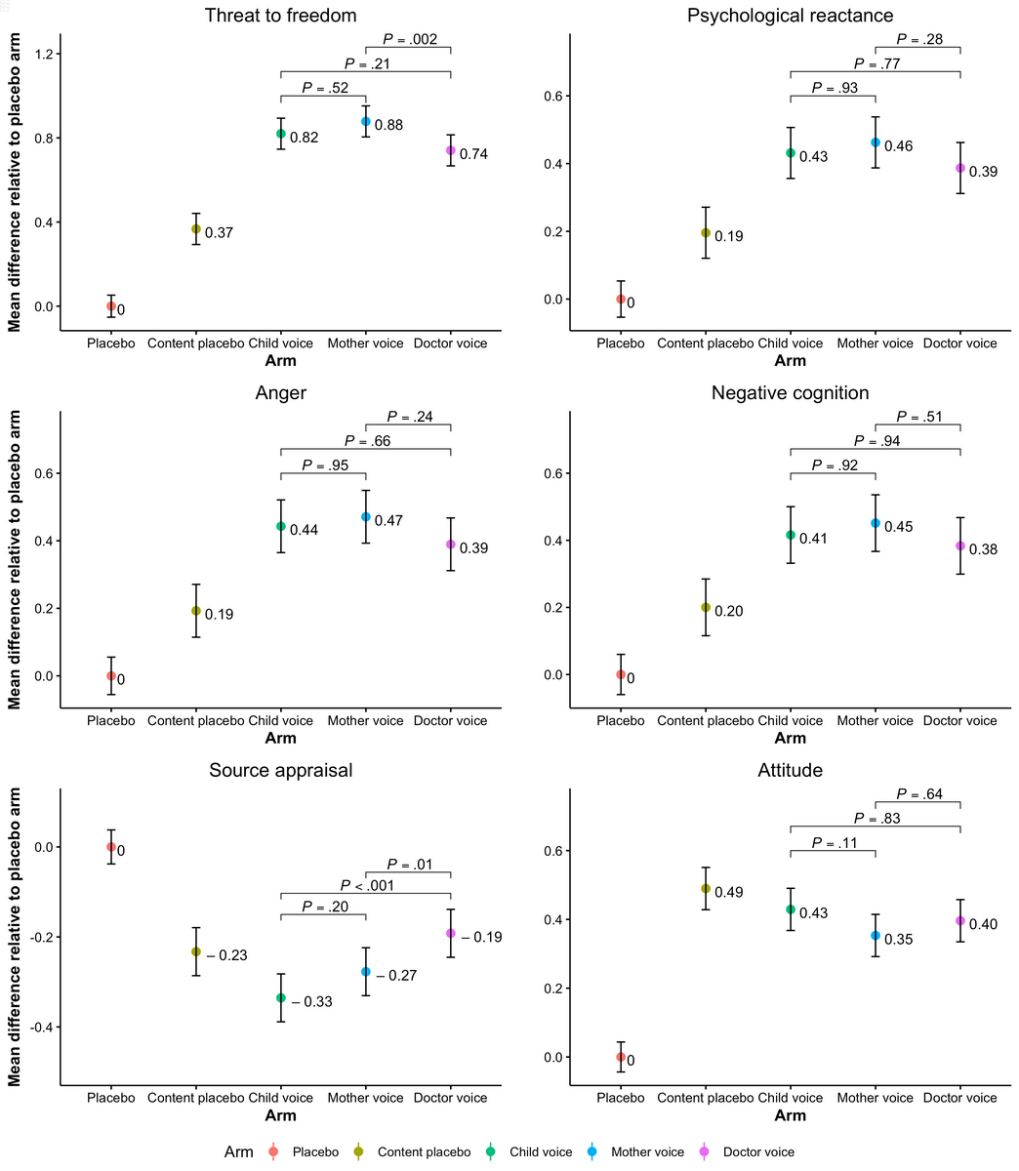
Mean differences in outcome measures among the placebo arm (reference arm), content placebo, and the 3 intervention arms (child, mother, and physician). *P* values represent the significance of the observed difference in means between the intervention arms.

Psychological reactance was assessed by measuring anger and negative cognition. Therefore, the average of all items on anger and negative cognition indicated the total score on reactance. Higher scores on anger implied a greater level of anger, whereas higher scores on negative cognition suggested a higher presence of negative thoughts following the video. Therefore, it was expected that higher scores on reactance would indicate higher levels of reactance triggered by the video. A 5-group comparison revealed a significant difference in the reactance levels (*P*<.001). Figure 3 shows that when compared with the placebo arm, the content placebo and intervention arms had significantly higher scores on reactance (*P*<.001). However, there was no statistically significant difference between intervention arms, suggesting that participants experienced the same amount of reactance when watching the video narrated by either the child, mother, or physician. When considering anger and negative cognition scales separately, the analysis revealed similar outcomes, where all arms were significantly different when compared with the placebo arm (Figure 3) and the intervention arms did not differ from each other (Figure 4).

### Source Appraisal

The evaluation of the message source was considered more favorable when the participants scored high on the source appraisal scale. The difference in means was significant between all the study arms (*P*<.001). As seen in Figure 3, the pairwise comparisons generated significant differences in mean scores between placebo and content placebo (*P*<.001) as well as placebo and intervention arms (<.001), suggesting that participants in the placebo arm had a more favorable evaluation of the video narrator than the participants in other study arms. However, there was no significant difference in the appraisal of the source between the content placebo and intervention arms (P=.25). When comparing intervention arms separately, participants who watched the video narrated by the physician had higher scores, that is, a more positive evaluation of the source, than the participants who watched videos narrated by the child (*P*<.001) or the mother (P=.01; Figure 4).

**Figure 5:**
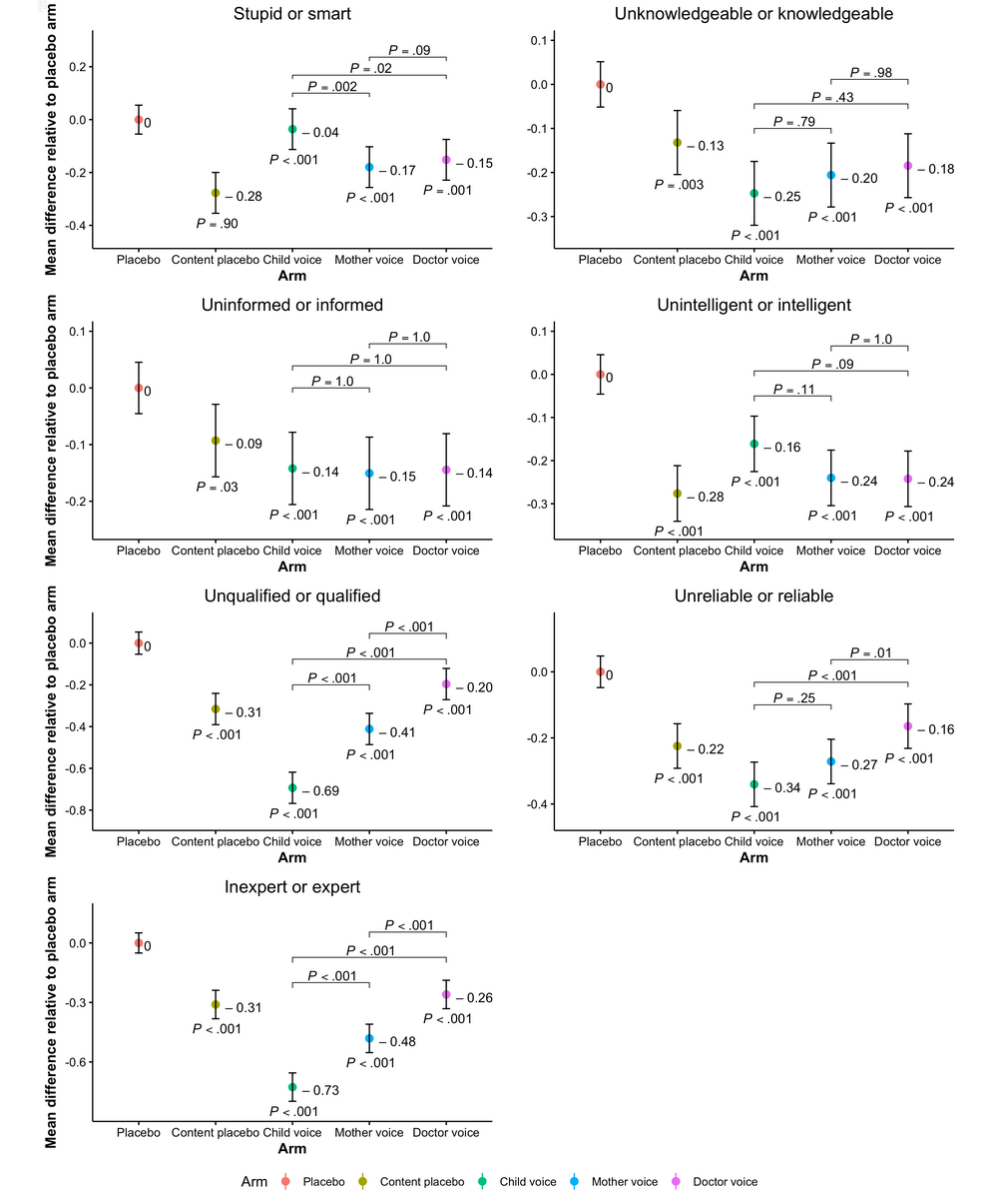
Source appraisal scale items. The y-axis shows the mean difference with CIs of the items in the content placebo, child, mother, and doctor arms relative to the placebo arm (reference arm). The x-axis shows the trial arms. *P* values under CIs represent the significance of the observed difference in means relative to the placebo arm, and *P* values over brackets represent the significance of the observed difference in means among the intervention arms.

Items on the source appraisal scale were also analyzed separately for a more detailed understanding of the message source evaluation as source (narrator) is the major component of our research question. As seen in Figure 5, the child narrator was considered to be smarter than mother (*P*=.002) and physician (*P*=.03). In addition, unlike the narrators in the content placebo (*P*<.001), mother (*P*<.001), and doctor (*P*<.001) arms, the narrator in the child arm was found to be as smart as the narrator in the placebo arm (*P*=.89). However, all 3 intervention arms (*P*<.001) and the content placebo arm (*P*=.003) scored significantly lower on the unknowledgeable or knowledgeable item in comparison with the placebo arm. There were no significant differences between intervention arms on this item. The placebo arm narrator was also seen to be more informed than the narrators in the content placebo (*P*=.04) and intervention arms (*P*<.001; Figure 5). The narrator in the placebo arm was also believed to be more intelligent, qualified, reliable, and expert than the narrators in the other 4 arms (*P*<.001). When comparing the intervention arms with each other, all narrators were considered to be equally informed and intelligent. However, the participants in the doctor arm rated the message source to be more qualified (*P*<.001), reliable (*P*<.001 and *P*=.02), and expert (*P*<.001) than those in the child and mother arms, respectively. Furthermore, the mother narrator was also seen as more qualified (*P*<.001) and expert (*P*<.001) than the child narrator, although the same difference was not found for the unreliable or reliable item (*P*=.25; Figure 5).

### Attitude

Higher scores on the attitude scale suggested that participants had a more favorable attitude toward the message advocacy. After the analysis showed a significant difference on the attitude score between the study arms (*P*<.001), pairwise comparisons confirmed that participants in the content placebo and intervention arms had significantly more positive attitudes toward the message when compared with participants in the placebo arm (*P*<.001; Figure 3). Furthermore, compared with the content placebo, participants in the intervention arm had significantly less favorable attitude toward the sugar videos (*P*<.001). As the message in all 3 intervention videos was the same, there were no statistically significant differences between the child, mother, and doctor arms (Figure 4).

## Discussion

### Principal Findings

In recent years, the use of a narrative-structured format and video-based animation has enabled the creative use of nonhuman and nonadult characters to promote persuasive health messages [38]. To further explore the design of effective health communication interventions and increase their long-term effects, we created a SAS video that can engage global audiences in evidence-based health promotion and be rapidly distributed on various social media platforms. In this study, we evaluated whether a SAS video about sugar intake could attenuate reactance to a health message (hypothesis 1) and compared the effectiveness of a child narrator, her mother, and physician in reducing reactance to a message about added sugar consumption (hypothesis 2).

We found that our SAS video aroused higher levels of reactance when compared with a content placebo video about sunscreen use (containing no sugar message) and a placebo video about earthquakes (containing no health message). With respect to our first hypothesis, we therefore demonstrate a causal relationship between exposure to a SAS video and the antecedents and components of reactance. In particular, our results show that compared with the placebo video, the content placebo video was perceived as more threatening, while the intervention videos were seen as the most threatening. In addition, participants who watched the intervention videos experienced significantly higher levels of anger and negative cognition than those in the placebo and content placebo arms. Although psychological reactance has not been fully tested in the context of digital health promotion, this study contributes evidence to the existing literature on persuasion and reactance [39].

One plausible explanation for the higher levels of reactance to the intervention videos may be rooted in the part that describes the death of the child’s father, which is attributed to the regular consumption of soda drinks. Some scholars have argued that health promotion messages can be effective when they increase people’s fear or concern about risky behaviors that can threaten their health [40,41]. In other words, when presented with fear, individuals may be more motivated to change their behaviors. The cognitive functional model by Nabi [42] suggests that before creating a health message, authors should determine which emotion would be most suited to their persuasive goals, adding that fear might be best used for preventing behaviors that lead to severe consequences, such as death. However, participants may have perceived this part of the story as an emotional manipulation and recognized the actual persuasive intention in the message. Some scholars have concluded that noticing a covert attempt to promote healthy behavior disguised as entertainment results in reactance, whereas a more direct persuasive attempt does not [7]. Several other studies [43,44] have also shown that health advertising material that was perceived to be manipulative caused more resistance and anger and was, therefore, less effective in changing attitudes. Taking these points into account, the removal of the emotional part of the video, where the death of the child’s father is portrayed, could potentially induce less reactance and negative attitudes toward the message. This presents a possible avenue for future research, in which we could compare videos with and without this emotional subplot.

In this study, we focused on 1 modifiable component—the social authority of the narrator—and its effect on reactance to a message about reducing sugar intake. Initially, we assumed that a child narrator would be a more persuasive messenger and would be less likely to arouse reactance when compared with the adult narrators (mother and physician), as the audience may view the child as nonthreatening and lacking vested interest. Contrary to our expectations, we found no evidence that the child narrator attenuated reactance when compared with the same intervention videos narrated by the mother and the family physician. Therefore, we fail to reject the null hypothesis of H2 in that there were no significant differences between the child and the mother and the child and the physician with respect to the threat to freedom, anger, negative cognition, and the combination of the last 2 components (state reactance).

However, one of the few significant differences that we observed was in the source appraisal component, which plays a critical role in this context. Our results show that the physician narrator was perceived to be more qualified and reliable and has more expertise than the child and the mother narrators. This is an expected finding as doctors are generally seen as experts in the health field and reliable sources of accurate and valid information. Earlier studies have shown that message recipients tend to be more motivated to change and persuaded by an expert rather than a non–expert message source [45,46]. However, it has also been suggested that the position of the person toward the message, that is, whether it is viewed as consistent with or discrepant from one’s current attitude toward the issue, as well as issue relevancy determine the persuasive effect of the message, regardless of the source expertise [47]. One study [48] found that when participants had low relevance to the issue, higher source expertise produced better attitudes toward the argument, even when the message quality was manipulated. However, when the message had high relevance, message quality had the biggest impact on the attitudes of the participants, whereas source expertise became a less important factor of persuasion. Our study provides further evidence to these findings as the videos narrated by child, mother, and physician produced similar reactance outcomes, suggesting that the message itself and its relevance may play a bigger role than the source of the message.

Another minor yet interesting finding was that the participants evaluated the child source as smarter than the mother and the physician. A possible explanation for this difference might lie in the fact that participants, who were exclusively adults, were not willing to call the child narrator stupid owing to potential social desirability bias, which is described as the tendency of research participants to give socially desirable responses instead of honest responses [49]. As we could not find any prior studies that compared child narrators to adult narrators, there is no evidence to support this assumption.

We do not believe that design differences between the intervention and placebo videos can account for differences in reactance across the trial arms. This is because we were careful to select placebo videos that were similar to the sugar intervention video, such that all videos were short (3.42 minutes), animated, story-based, and in English. The only systematic difference among the videos was the content of the narrated messages (about earthquakes, sunscreen, and sugar), which were the trial arms. Importantly, as we explain in Figure 1, the antecedents to reactance are *threat to freedom* and *trait proneness to reactance*. This means that design differences such as animation style, background shapes or colors, and target audience are not hypothesized to arouse reactance and hence are unlikely to account for differences in reactance. Nevertheless, we acknowledge that the sugar intervention video was narrated by a female voice, and the placebo videos were narrated by male voices. There is evidence that men are perceived as more credible than women, and women are perceived as more trustworthy than men [50]. To the best of our knowledge, there is no evidence that links the gender of the narrator to differences in reactance, which could be a future avenue of research.

The findings of this study make an important contribution to the literature on digital health promotion. Several studies that focused on added sugar reduction have used nonanimated, web-based videos, such as a puppet show [51], expert opinion intercut with case studies [52], video courses [53], and storytelling interviews [54]. Unlike the SAS video in our study, these interventions were approximately 6 to 15 minutes long, which is longer than the optimal time required for a social media format and focused on certain demographic groups and populations. Although we could not support the proposed hypothesis that a child can be a powerful and persuasive health promotion agent, our findings indicate that the quality and design of the health message should be considered more carefully in persuasive health promotion. The finding that a message with a persuasive intent, even when masked, provokes some kind of reactance may be reasonable, but the end goal of health promotion experts should be to create and promote SAS videos that would lead to a minimum amount of reactance and be almost comparable with a message in which persuasion is absent. The avoidance of intense emotional appeal and the use of narrative-based messages could be potentially successful components in health message design.

### Strengths and Limitations

This study had several strengths. First, we used an RCT design, which allowed us to eliminate any potential sources of bias by randomization. Randomization ensures that there are no systematic differences introduced at the enrollment stage. Second, the web-based nature of our experiment enabled us to reach a large sample size, which ensured the quality and reliability of the sample. The use of content placebo and placebo videos is also an innovative feature of our study, which enabled us to isolate the health awareness effect and content effect of the intervention video. We are not aware of any previous study that had such a large sample size and used a similar experimental approach to examine the social authority of the health message source. Once the design of the SAS video that we created is further examined and modified, it can be used for larger audiences on social media and other educational sources, as it is short, simple, and quickly scalable.

Our study has several limitations. First, Prolific uses convenience sampling to recruit participants, so that study places are filled on a first-come, first-serve basis. Thus, a considerable portion of responses could come from participants who are on the web at the time a study is launched or immediately afterward. Rapid-responder bias may be an issue if the required sample is very small or very specific. However, our study was general (men, women, other; aged 18-59 years; of any education level if a UK resident) and ran continuously for 3 consecutive days. In addition, Prolific has several mechanisms to reduce rapid-responder bias and equally distribute study places among active participants. For example, when a study is launched, Prolific sends an email to a random subset of all eligible participants every 48 hours until the sample size is reached. Therefore, it is unlikely that rapid-responder bias will have significantly affected our results. Second, our study had a sampling bias toward women (60.85%, 2442/4013 females vs 38.62%, 1550/4013 males) and participants with higher education (83.20%, 3339/4013) had a bachelor’s degree or higher). Similar sample distributions have been reported in several web-based studies [55,56]. It has been observed that most participant pools in the social sciences are biased toward Western, educated, industrialized, rich, and democratic individuals, as they are predominantly from the United Kingdom, United States, or Europe [57]. The generalizability of our findings may therefore be limited to the UK and the US contexts, and possibly to Europe. Further research should be conducted in other settings to make the results more generalizable to other geographies and cultures.

A third limitation is that the Prolific participants may have chosen to participate in our study because of the sugar-related topic. However, it is unlikely that this type of selection bias would have considerably affected our results for one important reason: randomization. For our RCT, we randomized participants to either the sugar intervention video, the content placebo video, or the placebo video, so that any topic-specific selection bias would have been uniformly distributed across the trial arms. Fourth, it is possible that participants may have been motivated by financial rewards, which could introduce a selection bias. Again, this form of bias would have been equally distributed across the trial arms because of our randomized design. In addition, financial rewards are standard for web-based studies and are acceptable when the research is not focused on a specific disease or treatment and does not involve potential risks [58]. The study reward was also relatively small (£1; US $1.37) and most of the participants were highly educated (most had a bachelor’s degree or higher), making it unlikely that participation was motivated by economic disadvantage. Indeed, previous research has reported that web-based research participants are motivated by a variety of reasons other than financial rewards, such as self-improvement, microtasking to avoid wasted time, and other emotional benefits [58]. Overall, it is unlikely that a small financial reward led to biases that significantly affected our results. Finally, although this was beyond the scope of our study, we acknowledge that further research is needed to determine if SAS video interventions for social media can be more cost-effective than mobile health or other nondigital approaches [59].

Taken together, the findings of this study demonstrate that the content of health messages may have a greater impact on reactance than the source of the message and its authority. Moreover, the use of SAS videos on social media can facilitate public health efforts to promote healthy behaviors to a larger audience. The experimental design and the use of multiple placebo groups in this study present novel approaches for further investigation in this area. It is essential to gain a better understanding of the unique ways in which messages about healthy behaviors are processed and the resulting emotions and intentions to advance health communication strategies worldwide.

## Appendix

Multimedia Appendix 1 CONSORT-eHEALTH checklist (V 1.6.1).

## Abbreviations

RCT: randomized controlled trial
SAS: short and animated story-based
